# Identifying Urinary Tract Infection–Related Information in Home Care Nursing Notes

**DOI:** 10.1016/j.jamda.2020.12.010

**Published:** 2021-01-09

**Authors:** Kyungmi Woo, Victoria Adams, Paula Wilson, Li-heng Fu, Kenrick Cato, Sarah Collins Rossetti, Margaret McDonald, Jingjing Shang, Maxim Topaz

**Affiliations:** a College of Nursing, Seoul National University, Seoul, Republic of Korea; b Center for Home Care Policy & Research, Visiting Nurse Service of New York, New York, NY, USA; c Department of Biomedical Informatics, Columbia University, New York, NY, USA; d School of Nursing, Columbia University, New York, NY, USA; e Data Science Institute, Columbia University, New York, NY, USA

**Keywords:** Urinary tract infection, natural language processing, home care, hospitalization, nursing

## Abstract

**Objectives:**

Urinary tract infection (UTI) is common in home care but not easily captured with standard assessment. This study aimed to examine the value of nursing notes in detecting UTI signs and symptoms in home care.

**Design:**

The study developed a natural language processing (NLP) algorithm to automatically identify UTI-related information in nursing notes.

**Setting and Participants:**

Home care visit notes (n = 1,149,586) and care coordination notes (n = 1,461,171) for 89,459 patients treated in the largest nonprofit home care agency in the United States during 2014.

**Measures:**

We generated 6 categories of UTI-related information from literature and used the Unified Medical Language System (UMLS) to identify a preliminary list of terms. The NLP algorithm was tested on a gold standard set of 300 clinical notes annotated by clinical experts. We used structured Outcome and Assessment Information Set data to extract the frequency of UTI-related emergency department (ED) visits or hospitalizations and explored time-patterns in documentation of UTI-related information.

**Results:**

The NLP system achieved very good overall performance (F measure = 0.9, 95% CI: 0.87–0.93) based on the test results obtained by using the notes for patients admitted to the ED or hospital due to UTI. UTI-related information was significantly more prevalent (*P* < .01 for all the tests) in home care episodes with UTI-related ED admission or hospitalization vs the general patient population; 81% of home care episodes with UTI-related hospitalization or ED admission had at least 1 category of UTI-related information vs 21.6% among episodes without UTI-related hospitalization or ED admission. Frequency of UTI-related information documentation increased in advance of UTI-related hospitalization or ED admission, peaking within a few days before the event.

**Conclusions and Implications:**

Information in nursing notes is often overlooked by stakeholders and not integrated into predictive modeling for decision-making support, but our findings highlight their value in early risk identification and care guidance. Health care administrators should consider using NLP to extract clinical data from nursing notes to improve early detection and treatment, which may lead to quality improvement and cost reduction.

Home care is becoming an increasingly important care venue for older and clinically complex patients. In the United States, 3.4 million people received home care services in 2017,^1^ and these numbers are expected to grow as the population ages and inpatient stays become shorter.^2^ Nurses treat patients in their homes while providing a wide range of care services such as patient education, custodial care, and wound treatment. Home care nurses are often the first to recognize and report patient deterioration or the appearance of dangerous symptoms.

Infections are common in home care, estimated to contribute to rehospitalization of more than 21% of postsurgical home care recipients within 30 days of home care.^3^ Urinary tract infection (UTI) is one of the most common infections in home care, with about 1.5% (27,000) of older adults in home care diagnosed with a UTI in 2017.^1,4^ If UTI symptoms are recognized early and UTI is effectively treated, the infection can be resolved within a few days or weeks. However, delayed UTI diagnosis and treatment can result in serious and life-threatening complications, such as renal damage or sepsis.^5^ Thus, recognizing UTI signs and symptoms early is critical, but the role of home care nurses in facilitating UTI recognition and treatment remains understudied.

An emerging body of research is using data science methods to facilitate early recognition of infections. For example, several studies have used clinical data from inpatient electronic health records to identify infections early.^5–9^ A recent study applied machine learning to develop techniques for better predicting central line^–^associated bloodstream infection in hospitals.^8^ Another study used natural language processing (NLP) to extract respiratory and gastrointestinal infection information from free-text primary care records in Singapore.^8^ This study found symptoms suggesting these infections documented with more than 90% precision and recall. Another study used NLP to automatically identify mentions of surgical site infections in radiology reports and provided evidence supporting the use of NLP for early detection of infections.^6^ Although these studies report insightful results, they used data from inpatient settings. Inpatient data are recorded with much higher frequency than data in home care. For example, patient’s vital signs can be documented several times per minute and there are additional physician and nurse assessments.^10^ In comparison, documentation in home care often happens once in several days when nursing visits take place. Also, as much as 50% of all documentation is in a narrative format; nevertheless, little research in inpatient and outpatient settings have explored this rich data source.

NLP is being increasingly applied to uncover insights hidden in clinical narratives.^11^ NLP uses a set of diverse techniques to automatically process large bodies of clinical text and extract meaning.^11^ For example, researchers have used NLP to identify social risk factors (eg, alcohol and drug abuse),^12^ mentions of depression,^13^ and wound-related information.^14^ We have recently applied NLP to identify fall-related information in home care narrative notes.^15^ However, no previous NLP studies have focused on infections in home care, and the value of nursing home care narratives remains underexplored.

This study aimed (1) to create and validate an NLP algorithm to identify UTI-related information in home care clinical notes and (2) to compare the frequency of UTI-related information in the clinical notes among patients who had ED visits or were hospitalized for UTI during home care vs other patients who did not have such events.

## Methods

### Study Data Set

This study used a large corpus of home care visit notes (n = 1,149,586) and care coordination notes (n= 1,461,171) for 89,459 patients treated by clinicians of the largest nonprofit home care agency in the United States (located in New York, NY) during 2014. Because some patients were admitted to home care multiple times, the data set includes 112,237 unique episodes of care defined as a period of time from admission to discharge from home care services (usually a period of 30–60 days). Notes were completed by visiting home care nurses using the agency’s electronic health record after a patient visit. Visit notes ranged from lengthy admission notes to shorter progress notes. The average visit note length was about 150 words. Care coordination notes were shorter (with average length of about 50 words) and often described issues encountered during patient care, such as needed equipment, follow-up on patient symptoms, and need in therapy.

### UTI-Related Information Model Development

Our first step was creating an information model describing any information we would like our NLP system to extract automatically from clinical notes. To accomplish that, we first conducted a thorough literature search in research databases [eg, PubMed, Google Scholar, the Cumulative Index to Nursing and Allied Health Literature (CINAHL)] to identify studies of UTI incidence and treatments. We used this literature and our clinical expertise in home care to generate a list of 6 categories of UTI-related information that we would like our NLP system to be able to find automatically, including (1) UTI-specific names, (2) UTI-specific symptoms, (3) UTI-nonspecific symptoms, (4) fever, (5) nausea/vomiting, and (6) confusion. UTI-specific symptoms included hematuria, dysuria, and pyuria, whereas UTI-nonspecific symptoms included abdominal pain, pelvic discomfort, and supra-pubic pain.

Next, we used a large-scale, standardized health terminology called the Unified Medical Language System (UMLS)^16^ to identify a preliminary list of terms for each of the 6 categories. UMLS links many other terminologies [eg, the Systematized Nomenclature of Medicine–Clinical Terms (SNOMED-CT),^17^ the International Statistical Classification of Diseases and Related Health Problems (ICD),^18^ the International Classification for Nursing Practice (ICNP)^19^] and compiles lists of relations, including synonyms from multiple terminologies. For example, UMLS’s concept “Urinary tract infection” (UMLS ID C0041029) has 16 unique synonyms, including “Urinary tract infectious disease,” “Tract, Infection Of Urinary,” “Urinary tract infection, NOS.” The preliminary list of terms for the UTI names category also included synonyms for pyelonephritis, urethritis, and cystitis (n=43 prepopulated synonyms). We extracted lists of UMLS synonyms for each of the 6 categories of UTI-related information model.

### NLP System Development and Validation

**Fig. 1.**
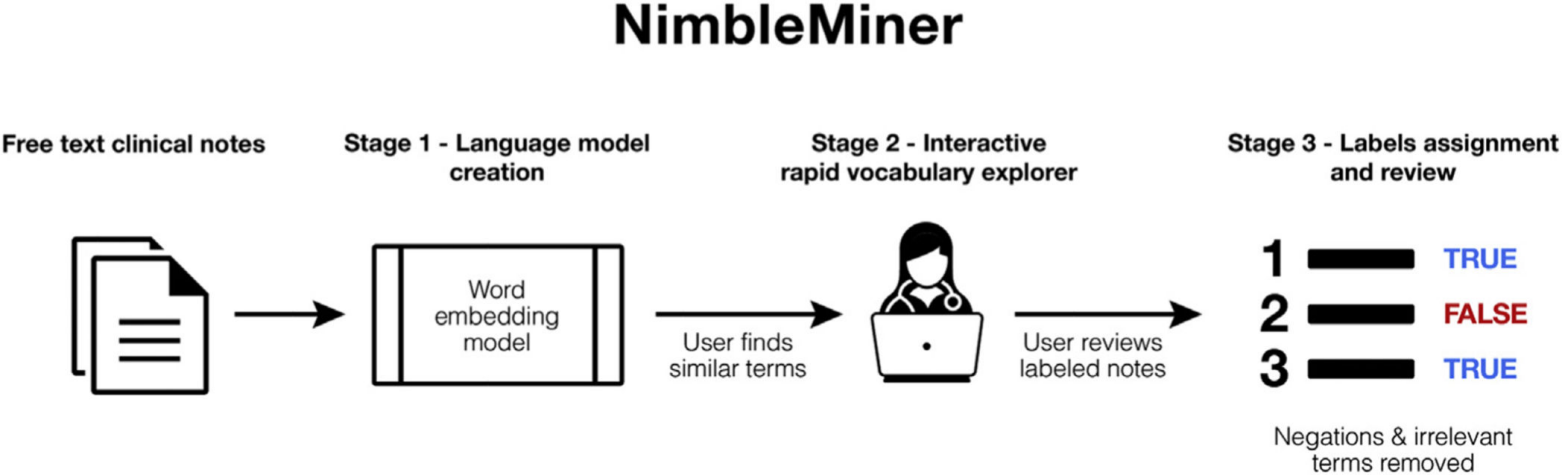
NimbleMiner architecture.

We used NimbleMiner to develop the NLP algorithms. Nimble-Miner is a user-driven text classification system previously applied in different domains,^20,21^ including home care.^15^ NimbleMiner includes several stages of clinical note processing that are briefly described here (Figure 1), with more details provided in Topaz et al.^22^

### NimbleMiner System Architecture

#### Stage 1: language model creation

Language models are statistical representations of a certain body of text–in our case, clinical notes. To create a language model in NimbleMiner, the user is required to identify a large corpus of clinical notes (a file that includes all the notes). We use a specific type of language model called a word embedding model^23^ because this model is most appropriate for our tasks.

#### Stage 2: interactive rapid vocabulary explorer

**Fig. 2.**
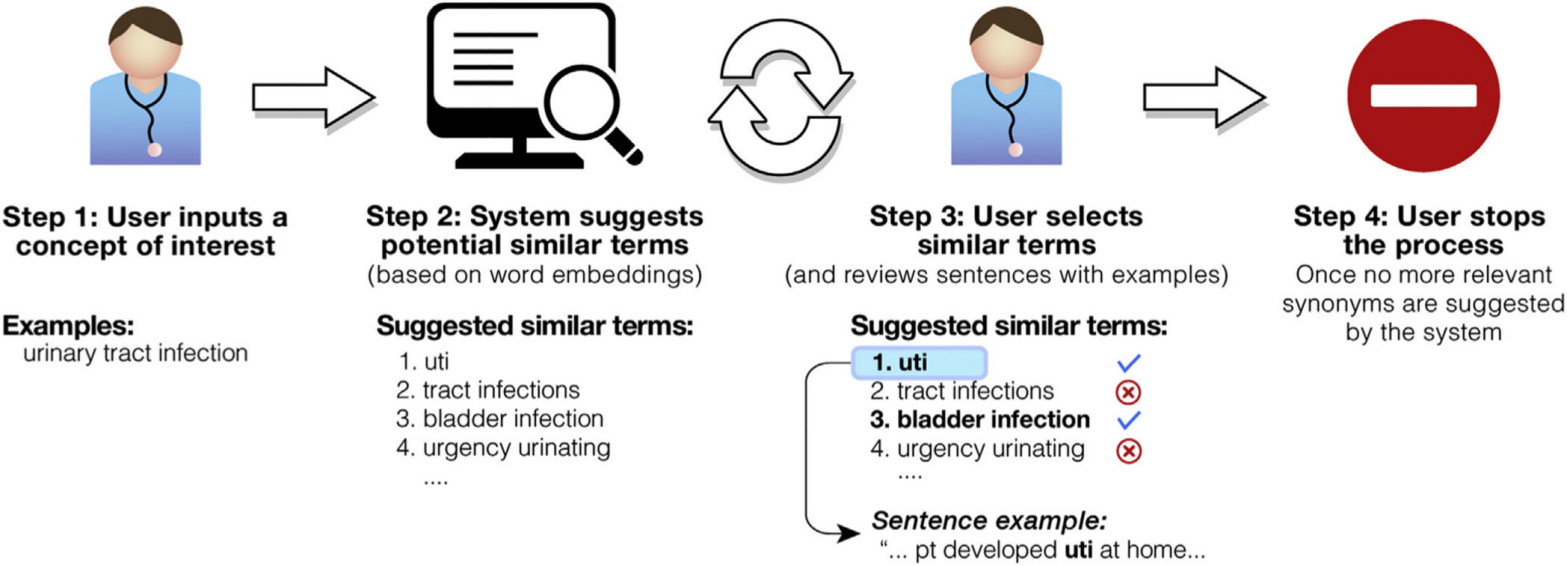
Interactive rapid vocabulary explorer.

This stage is designed to help users rapidly discover large vocabularies of relevant terms and expressions. The user enters a query term of interest (eg, “urinary tract infection”), and the system returns a list of similar terms it identified as relevant (eg, “uti,” “tract infections,” “bladder infection”). In our case, we prepopulated lists of similar expressions for each of the 6 UTI-related information categories extracted from the UMLS. The list of suggested similar terms is based on the cosine term similarity metric^23^ extracted from the word embedding model. The user selects and saves the relevant terms by clicking on them in the interactive vocabulary explorer screen. Negated terms (eg, “no uti,” “uti ruled out”) or other irrelevant terms (eg, “previous history of uti,” “questionable uti”) that are not selected by the user are also saved in the system for later tasks, such as negation detection. Figure 2 describes the steps of the vocabulary explorer stage.

#### Stage 3: label assignment and review

The system uses similar terms discovered by the user during stage 2 to assign labels to clinical notes (while excluding notes with negations and other irrelevant terms). Assigning a positive label means that a concept of interest is present in the clinical note. In our case, a positive label of UTI-specific symptom means that 1 or more of the symptoms are described in the clinical note. When needed, the user reviews and updates lists of similar terms and negated similar terms. The user reviews the clinical notes with assigned labels for accuracy. This weakly supervised rapid labeling approach is based on a positive label learning framework validated in previous research.^24,25^

NimbleMiner can be downloaded from http://github.com/mtopaz/NimbleMiner under GNU General Public License v3.0.

### Study Data Sets for Rapid Vocabulary Exploration

We experimented with 2 large collections of text documents for vocabulary exploration. First, we learned the language model using all ~2.6 million home care notes (n = 1,149,586 visit notes + n = 1,461,171 care coordination notes). We used this model as the baseline. Next, to potentially expand the vocabulary beyond language available in clinical notes, we downloaded a large collection of article abstracts from PubMed. To obtain the abstracts, we searched PubMed using a query term “urinary tract infection,” which resulted in 46,592 abstracts. These abstracts were downloaded and processed by NimbleMiner to create an additional PubMed UTI language model. Each model was queried independently by each of the 3 expert users [2 master’s-level registered nurses (RNs) with more than 10 years’ experience in home care and 1 PhD-level RN with expertise in informatics], and we calculated the number of additional similar terms discovered when the PubMed UTI model was added to the baseline model.

### NLP System Evaluation

We created a gold standard testing set of clinical notes using a high likelihood sampling approach as follows. First, we identified a subset of patients admitted to a hospital for UTI during a home care episode, based on the structured data. Among these patients, we extracted a random subset of 300 clinical notes (50% visit notes and 50% care coordination notes). Each note was annotated by 2 human expert reviewers (2 master’s-level RN with more than 10 years’ experience in home care) for the presence of 1 or more of the 6 UTI-related information categories. The interrater reliability was relatively high (Kappa statistics = .87), indicating strong agreement between reviewers.^26^ All disagreements were reviewed and adjudicated by another PhD-prepared RN.

Next, we applied our NLP system on the gold standard testing set and for each category calculated precision (defined as the number of true positives out of the total number of predicted positives), recall (the number of true positives out of actual number of positives), and *F* score (the weighted harmonic mean of the precision and recall). We implemented bootstrapping to provide more generalizable and robust NLP system performance results with 95% confidence intervals (CIs). Specifically, we calculated NLP performance metrics repeatedly 1000 times, where at each iteration we randomly sampled (with replacement) two-thirds of clinical notes (n=200) from the general sample of N=300 clinical notes. NLP system performance metrics were then averaged and reported with 95% CIs.

### Comparisons Based on Structured Data

We used structured data to compare the frequency of UTI-related information in the clinical notes among patients who were hospitalized for UTI during home care episode vs the general patient population. Home care nurses are required to document a reason for hospital or ED admission from home care (OASIS data set item M2430 and M2310, respectively), and we used this structured data field to identify a subpopulation of patients with UTI hospitalization. We split the patient sample into patients admitted to the ED or hospitalized from home care for UTI vs the rest of the patients. We compared the frequency of UTI-related information in the clinical notes for patients with UTI vs patients in the general sample. Institutional review board at the home care agency that provided the data approved the study protocol.

## Results

### Interactive Vocabulary Explorer

Every model was queried by each of the 3 expert users with substantial agreement on the included terms; on average, 73% of terms identified by each user in each category were also identified by 1 or both of the other 2 expert users. Compared with vocabularies extracted from standard terminologies, the baseline model was helpful in discovering between 42% and 606% more similar terms for the target categories (Supplementary Table 1). For almost all categories, querying the baseline model resulted in almost twice as much language being discovered compared with the PubMed model. The exception category for which more terms were discovered with the PubMed model was UTI-specific names. In general, the largest language expansions were observed using the baseline model for confusion (6-fold language expansion) and nausea/vomiting (4-fold language expansion).

### NLP System Performance

Overall, the difference between the systems built using the baseline and baseline + PubMed terms was minimal. Both NLP systems achieved very good average performance based on the test results obtained by using the notes for patients admitted to the ED or hospital due to UTI (average performance for all 6 categories, *F* measure = 0.9, 95% CI: 0.87–0.93), with the baseline + PubMed system achieving slightly better performance for the category of UTI-specific symptoms (Supplementary Table 2). In addition, the NLP system’s overall positive predictive value was 83.5% and the negative predictive value was 93.7%, both indicating high performance.

### Clinical Note Labeling Results

Next, we applied the NLP system to label all the clinical notes in the sample. When at least 1 UTI-related category was discovered, note labeling using the baseline + PubMed model resulted in 6.1% more episodes than the baseline-only model (Table 1).

Table 2 describes the number of notes with UTI terms by domain. UTI-related terms were significantly more frequent (*P* < .01 for all the tests) in home care episodes with UTI-related hospitalization or ED admission. The majority of home care episodes (81%) with UTI-related hospitalization or ED admission had at least 1 category of UTI-related terms vs 21.6% among episodes without such events.

### Frequency of Documentation

**Fig. 3.**
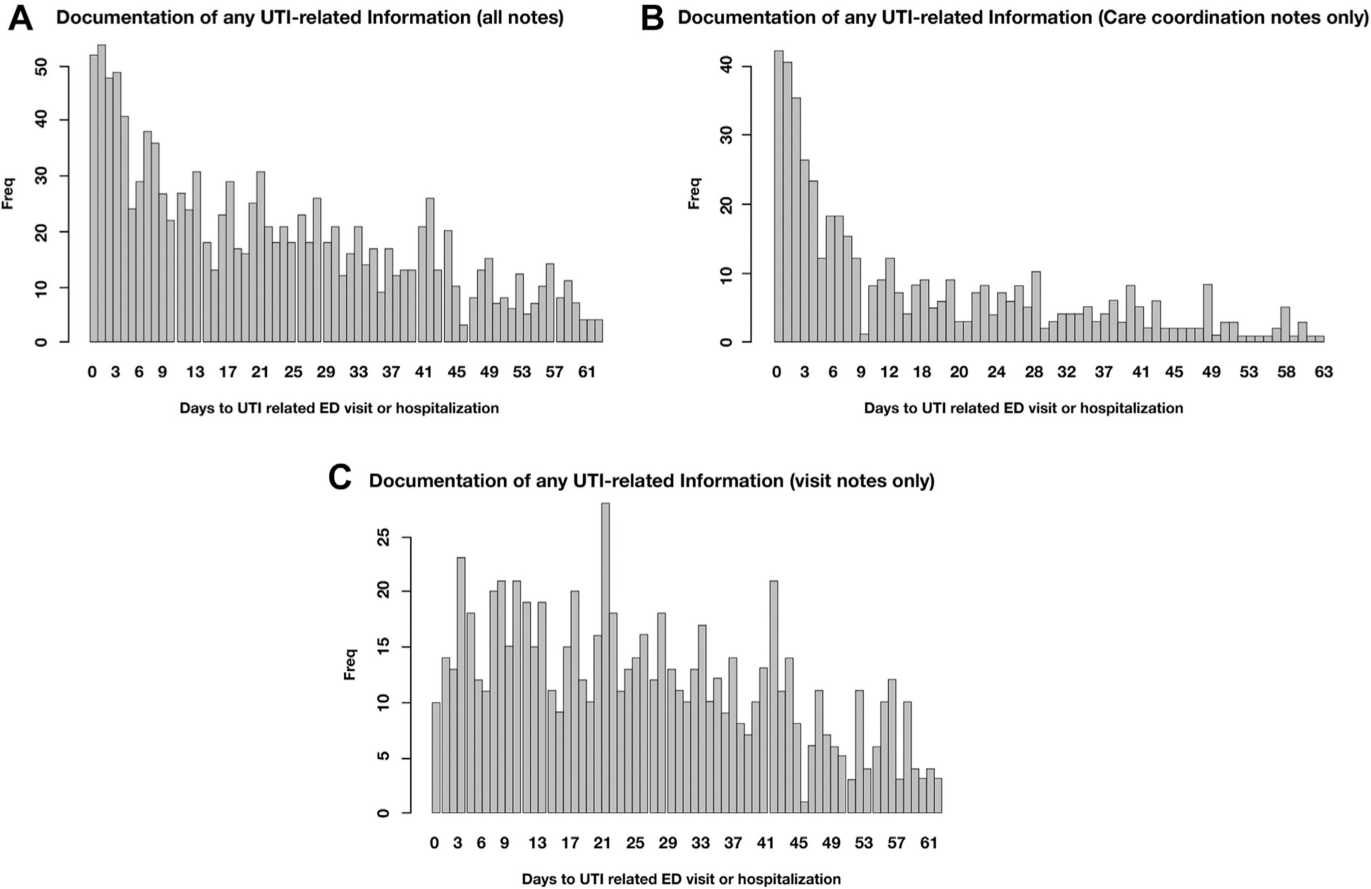
(A) Frequency of any UTI-related documentation (all notes) over time among patients admitted to ED or hospitalized with UTI. (B) Frequency of any UTI-related documentation (care coordination notes only) over time among patients admitted to ED or hospitalized with UTI. (C) Frequency of any UTI-related documentation (visit notes only) over time among patients admitted to ED or hospitalized with UTI.

Figure 3 describes the appearance of UTI-related information over time among home care episodes resulting in UTI-related hospitalization or ED admission. Figure 3A suggests that frequency of UTI-related information documentation increased in advance of UTI-related hospitalization or ED admission, peaking within a few days before the event. Figure 3B and C provide further insights and suggest that UTI-related information documented in care coordination notes had spiked within a few days before UTI-related hospitalization or ED admission (Figure 3B), whereas similar documentation in visit notes remained fairly stable over time (Figure 3C).

Similar trends were observed with UTI-specific and UTI-nonspecific symptoms. For details, please see Supplementary Figure 1. We also conducted a sensitivity analysis limiting the data to 1 home care episode per patient (first episode) to avoid potential inflation of certain observations. This sensitivity analysis did not identify any notable differences between the results.

## Discussion

We discovered significantly more terms and expressions relevant to UTI domains using models based on clinical notes and literature compared with terms extracted from standard terminologies. This finding is consistent with our previous work^22^; standard terminologies include lists of expert-curated standard terms, whereas clinical notes and literature might include a variety of lexical variants, such as abbreviations, misspellings, and lay language expression.

Querying the baseline model that included a large collection of home care notes resulted in up to 6-fold vocabulary expansion (ie, new terms or expressions added to the vocabulary) compared with standard terminologies. In comparison, querying an additional PubMed model resulted in a lower rate of language expansion for most UTI domains. One exception was an expansion of the UTI-specific names category. This expansion was largely due to diverse abbreviations used in the literature compared with clinical notes. Literature was abundant with terms such as RUTI (recurrent UTI), HAUTI (hospital acquired UTI), and LUTI (lower UTI). Overall, NLP systems implemented using the baseline or baseline + PubMed terms achieved very good performance when applied on a human expert–annotated gold standard data set of clinical notes. Similar to previous applications of NimbleMiner in other domains,^15,20,21^ these results prove the feasibility of accurate extraction of UTI-related language from clinical notes.

When at least 1 UTI-related category was discovered, labeling using the baseline + PubMed model resulted in 6.1% more episodes than when using just the baseline model. This finding highlights the added value of combining multiple sources for language discovery purposes. Relatively small language gains at the language discovery phase resulted in moderate labeling gains during the note-labeling phase.

About 1 in 5 clinical notes (21.6%) included UTI-relevant information. The most frequent category was UTI-specific names (65%) among episodes with UTI-related hospitalization or ED admission, whereas confusion (9.4%) was most common among episodes without UTI-related hospitalization or ED admission. These findings seem reasonable, because according to some estimates, about 20% of older adult patients in hospitals are confused or suffer from delirium,^24,25^ whereas confusion prevalence in home care settings remains unknown. It is important to note that confusion alone cannot be used as a symptom of UTI, though patients with confusion might be evaluated for other UTI-specific symptoms. Some of the other common categories mentioned in about 5% of the home care episodes were UTI-specific names, fever, and nausea/vomiting. These numbers are reasonable because home care patient population includes home-bound older adults who are likely to suffer from multiple symptoms and conditions.^15,27–29^

We found a significantly higher frequency of documentation of UTI terms among patients admitted to ED or hospitalized for UTI-related reasons. Four of 5 such patients had documentation of UTI-related information vs 1 in 5 patients in the general sample. Frequency of UTI-related information documentation increased in advance of UTI-related hospitalization or ED admission, peaking within a few days before the event. Other literature that is starting to explore nursing documentation content and patterns supports these findings. For example, a previous inpatient setting study indicated that increased frequency of nursing clinical assessments (reflected in documentation) serve as a proxy measure of when a nurse is concerned about a patient.^30^ Another study found that negative sentiments captured in hospital nursing notes are associated with outpatient mortality.^31^ Our study is the first to report similar results of increasing documentation patterns specific to UTI using NLP in home care.

Interestingly, the peak of UTI documentation before UTI-related health service use was most evident in care coordination notes compared with visit notes, where the documentation pattern remained fairly stable over time. The difference in documentation patterns might be explained by the nature of the notes; care coordination notes are often used to document communication with other providers, for example, consulting with primary care provider about patient symptoms (eg, “vn found blood in urine- will follow up with md”) or documenting a phone call to remind patient to follow-up with a physician (eg, “phone call with pt. pt to f/u with urology re: painful urination”). On the other hand, nursing visit notes are often used to document routine care over time rather than emerging concerning findings or urgent care needs. This finding shows the importance of examining different types of nursing notes separately for the NLP analysis, as the relevance and frequency of information included in these notes may vary.

The study findings have implications for clinical researchers and administrators interested in early detection of infections. Identifying patients who are starting to develop an infection early can allow for timely interventions that result in the prevention of related hospitalization or ED admissions, which is one of the major patient quality outcomes measured in home care.^32^ As alternative payment models focusing on “pay-for-performance” are becoming more common in inpatient and outpatient settings, the incentive to prevent rehospitalization and ED admissions through appropriate and efficient intervention and/or care coordination in home care will become stronger. For example, the Centers for Medicare & Medicaid Services (CMS) have recently introduced an alternative payment model, the Bundled Payments for Care Improvement Advanced (BPCI Advanced).^33^ BPCI Advanced is a voluntary payment program that allows home care agencies to receive additional funds to cover a beneficiary’s episode of care if quality is maintained, although the costs remain below a spending threshold. Home care agencies that reduce rehospitalizations and ED admissions, while controlling care costs through early detection of an infection, would qualify for the incentives. The NLP algorithm presented here may help to support these efforts.

The following hypothetical scenario describes a specific example of how an NLP algorithm can be applied to improve early detection and treatment of UTI in home care. We envision integrating the NLP algorithm into home care electronic health records in a way that all new clinical notes generated for all patients will be automatically screened for the presence of UTI-related information. Once a pattern of increased documentation of UTI-related information is identified, the electronic health record system will generate an alert that will be shared with a home care nurse, an infection specialist, or a care coordinator within the home care agency. The nurse will examine the clinical documentation to identify whether appropriate care was provided to the patient in response to concerning documentation patterns. Based on the nursing assessment, a patient might require additional testing or care, including receiving dipstick urinalysis testing or referral to a urologist. However, the effectiveness of such a hypothetical scenario remains to be tested in a clinical trial with comparison of this intervention with existing standards of care.

A point of caution–UTI misdiagnosis is common and, according to some reports, account for up to 50% of all UTI diagnoses.^34,35^ Our NLP algorithm can help to detect specific UTI symptoms, but clinicians need to be attentive in reviewing NLP findings in order to make correct diagnoses and consequently apply antibiotics. As with any decision-making support tool, clinicians’ critical review of supporting data is an important way to mitigate unnecessary antibiotic use, which is considered a potential adverse consequence of early UTI detection.^36,37^

This study has several important limitations. First, the study was conducted using data from 1 home care agency, and findings might be not widely generalizable. In addition, we did not explore the statistical significance of association between the UTI-related information in clinical notes and negative outcomes such as UTI-related ED admission or hospitalization or hospitalization for other reasons. Moreover, we did not include temporal terms in our language models. NLP system performance was tested on the clinical notes for patients admitted to the ED or hospital due to UTI. This limits our understanding of the performance of the NLP system on the notes for patients without UTI-related health service use. The NLP system applied here did not use machine learning approaches, such as neural networks, which might have resulted in better predictive performance. Finally, the reason for hospitalization as documented by nurses might sometimes be erroneous or incomplete, and further studies could explore using additional data sources, such as Centers for Medicare & Medicaid Services claims data on reasons for hospital admission, to verify our results.

## Conclusions and Implications

This study is the first to implement an NLP approach to identify UTI-related information in home care nursing notes. We found a significantly higher frequency of UTI-related information documentation among patients admitted to ED or hospitalized for UTI-related reasons, with frequency peaking within a few days before the UTI-related care event. This provides valuable data to inform possible early interventions (eg, timely referral and management prior to emergency care). Nursing documentation is often overlooked by stakeholders and is not integrated into machine learning algorithms for predicting critical health outcomes. Our findings highlight the potential use of nursing assessments and documentation for early identification of patients at risk and guiding care management improvement. We recommend further studies that examine the effectiveness of applying advanced data-mining technology, for example, NLP, to extract clinical data from nursing notes in order to improve early detection and treatment, which can in turn improve care quality and reduce costs.

## Supplementary Material

1

## Figures and Tables

**Table 1:** Clinical Notes Labeling Results

	Care Coordination Notes, n (%) (n = 1,461,171)	Visit Notes, n (%) (n = 1,149,586)	Total Home Care Episodes With UTI Terms, n (%) (n = 112,237)
No. of notes with UTI terms using the baseline model*	9967 (0.7%)	35,932 (3.1%)	
No. of home care episodes with UTI terms using the baseline model	6901 (0.5%)	21,539 (1.9%)	23,418 (20.9%)
No. of notes with UTI terms using the baseline + PubMed model^†^	10,484 (0.7%)	37,424 (3.3%)	
No. of home care episodes with UTI terms using the baseline + PubMed model	7179 (0.5%)	22,259 (1.9%)	24,841 (22.1%)^‡^

* Baseline model: UTI language model developed by NLP program (NimbleMiner) using approximately 2.6 million home care notes.

† PubMed model: UTI language model developed by NLP program (NimbleMiner) using PubMed abstracts (n = 46,592).

‡ % additional home care episodes with UTI terms found using the baseline + PubMed model (compared to baseline only model) = 6.1%.

**Table 2:** Clinical Notes With UTI Terms by Domain

UTI Terms by Domain	Home Care Episodes Without UTI-Related Hospitalization or ED Admission (n = 111,644)		Home Care Episodes With UTI-Related Hospitalization or ED Admission (n = 593)*	
	n	%	n	%
Any UTI-related category	24,208	21.6	482	81.3
UTI-specific names	5354	4.8	384	64.8
UTI-specific symptoms	2335	2.1	139	23.4
UTI-nonspecific symptoms	2524	2.2	40	6.7
Nausea and vomiting	5194	4.6	73	12.3
Fever	4876	4.3	141	23.8
Confusion	10,590	9.4	148	25.0

* All differences in proportions between home care episodes with UTI-related hospitalization or ED admission vs without UTI-related hospitalization or ED admission were statistically significant (*P* < .01).
