# Supplementary material for: Identifying Urinary Tract Infection–Related Information in Home Care Nursing Notes
Source: J Am Med Dir Assoc. Author manuscript; Available in PMC 2021 May 9. (PMC8106637; doi:10.1016/j.jamda.2020.12.010)
Supplement: 1 [file NIHMS1656905-supplement-1.pdf]

ARTICLE IN PRESS

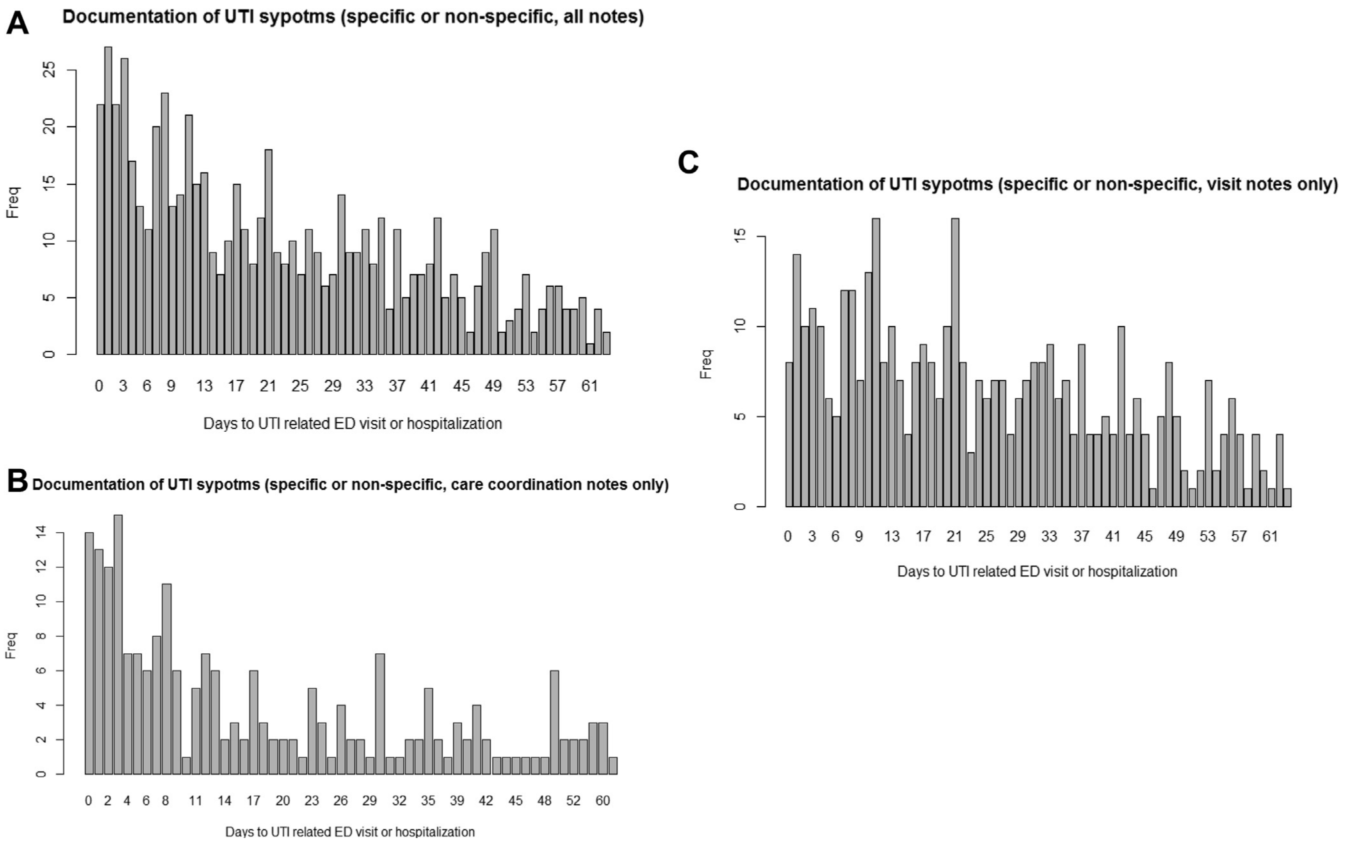

Supplementary Fig. 1. Frequency of documentation of any UTI-specific and UTI-nonspecific symptoms over time among patients who were admitted to ED or hospitalized with UTI: (A) all notes; (B) care coordination notes only; (C) visit notes only. Frequency of UTI-specific and UTI-nonspecific symptoms documentation increased close to UTI-related hospitalization or ED admission, peaking within a few days before the event (A-C). UTI-specific and UTI-nonspecific symptoms documented in care coordination notes had spiked within a few days before UTI-related hospitalization or ED admission (B), whereas similar documentation in visit notes remained fairly stable over time (C). ED, emergency department; UTI, urinary tract infection.

Supplementary Table 1
Vocabulary Discovery by Different Language Models

	UTI-Specific Names	UTI-Specific Symptoms	UTI-Nonspecific Symptoms	Nausea and Vomiting	Fever	Confusion
No. of similar terms prepopulated from standard terminologies	43	89	49	34	35	31
No. of similar terms added from baseline model*	18	66	38	143	79	188
% of similar terms added to prepopulated similar terms	42	74	78	421	226	606
No. of similar terms added from PubMed model†	52	28	12	99	44	130
% of similar terms added to prepopulated similar terms	121	31	24	291	126	419
Total unique similar terms (all models)	113	183	99	276	158	349

*Baseline model: UTI language model developed by NLP program (NimbleMiner) using ~2.6 million home care notes.
†PubMed model: UTI language model developed by NLP program (NimbleMiner) using PubMed abstracts (n=46,592).

Supplementary Table 2
NLP System Performance With 95% CI

	All Simclins					
	Recall	(95% CI)	Precision	(95% CI)	*F* Measure	(95% CI)
UTI names	0.95	(0.94, 0.96)	0.91	(0.90, 0.92)	0.93	(0.89, 0.97)
UTI-specific symptoms	0.77	(0.73, 0.81)	0.74	(0.71, 0.77)	0.75	(0.74, 0.77)
UTI-nonspecific symptoms	0.71	(0.69, 0.73)	0.78	(0.76, 0.80)	0.74	(0.73, 0.75)
Nausea and vomiting	0.87	(0.85, 0.89)	0.91	(0.88, 0.94)	0.89	(0.85, 0.93)
Fever	0.94	(0.91, 0.97)	0.93	(0.92, 0.94)	0.93	(0.92, 0.95)
Confusion	0.83	(0.83, 0.83)	0.82	(0.80, 0.84)	0.82	(0.78, 0.87)
All categories	0.89	(0.86, 0.92)	0.91	(0.88, 0.94)	0.90	(0.87, 0.93)
